# Protein aggregation in amyotrophic lateral sclerosis

**DOI:** 10.1007/s00401-013-1125-6

**Published:** 2013-05-15

**Authors:** Anna M. Blokhuis, Ewout J. N. Groen, Max Koppers, Leonard H. van den Berg, R. Jeroen Pasterkamp

**Affiliations:** 1 Department of Neuroscience and Pharmacology, Rudolf Magnus Institute of Neuroscience, University Medical Center, Utrecht, The Netherlands; 2 Department of Neurology and Neurosurgery, Rudolf Magnus Institute of Neuroscience, University Medical Center, Utrecht, The Netherlands

**Keywords:** Amyotrophic lateral sclerosis (ALS), Aggregation, Protein degradation, Motor neuron, RNA granule

## Abstract

Amyotrophic lateral sclerosis (ALS) is a neurodegenerative disease characterized by the aggregation of ubiquitinated proteins in affected motor neurons. Recent studies have identified several new molecular constituents of ALS-linked cellular aggregates, including FUS, TDP-43, OPTN, UBQLN2 and the translational product of intronic repeats in the gene *C9ORF72*. Mutations in the genes encoding these proteins are found in a subgroup of ALS patients and segregate with disease in familial cases, indicating a causal relationship with disease pathogenesis. Furthermore, these proteins are often detected in aggregates of non-mutation carriers and those observed in other neurodegenerative disorders, supporting a widespread role in neuronal degeneration. The molecular characteristics and distribution of different types of protein aggregates in ALS can be linked to specific genetic alterations and shows a remarkable overlap hinting at a convergence of underlying cellular processes and pathological effects. Thus far, self-aggregating properties of prion-like domains, altered RNA granule formation and dysfunction of the protein quality control system have been suggested to contribute to protein aggregation in ALS. The precise pathological effects of protein aggregation remain largely unknown, but experimental evidence hints at both gain- and loss-of-function mechanisms. Here, we discuss recent advances in our understanding of the molecular make-up, formation, and mechanism-of-action of protein aggregates in ALS. Further insight into protein aggregation will not only deepen our understanding of ALS pathogenesis but also may provide novel avenues for therapeutic intervention.

**Electronic supplementary material:**

The online version of this article (doi:10.1007/s00401-013-1125-6) contains supplementary material, which is available to authorized users.

## Introduction

Amyotrophic lateral sclerosis (ALS) is a fatal neurodegenerative disease caused by the loss of both upper and lower motor neurons. Affected patients develop progressive muscle weakness eventually leading to death due to respiratory failure, typically 3–5 years after symptom onset. ALS affects ~2 out of 100,000 individuals per year [76]. In the majority of patients the disease occurs sporadic and is referred to as sporadic ALS (SALS). In 5 % of cases there is a family history of ALS (FALS) [29]. The presence of protein aggregates in affected motor neurons is a characteristic, but still poorly understood hallmark of SALS and FALS patients. Recently, many new ALS causing gene defects have been identified including mutations in *HNRNPA1*, *PFN1*, *C9ORF72*, *UBQLN2*, *OPTN*, *VCP,* *FUS* and *TARDBP* [1, 105]. Most of these mutations are rare and cause ALS in a small subgroup of patients. Remarkably, however, the proteins encoded by these genes are present in protein aggregates of a large proportion of non-mutation carriers indicating a more widespread role for their abnormal localization in ALS pathogenesis (Table 1). Moreover, some of these proteins are present in pathological aggregates of other neurodegenerative disorders such as frontotemporal lobar degeneration (FTLD), spinocerebellar ataxia (SCA), Huntington’s disease, Alzheimer’s disease and inclusion body myositis (IBM), indicating a more general involvement in neurodegeneration.

**Table 1:** Overview of the molecular composition of ALS aggregates

	Ub	p62	SOD1	TDP-43	FUS	OPTN	UBQLN2	ATXN2	C9ORF72	Other proteins	RNA granule markers	Colocalization in same inclusion shown in ALS
SALS	+	+	−	+	±	+	+	+	−	VCP [96] TAF15 [39] PDI [55] RGNEF, Peripherin, pNFH [103] RBM45 [36]	TIA-1, eIF3, XRN1, Staufen [19, 131, 200]	TDP-43 and FUS [103] FUS and ATXN2 [56] OPTN and TDP-43 [135] TDP-43 and PABP1 and TIA-1 [19, 131, 200] TDP-43 and RBM45 [36]
ALS SOD1	+	+	+	−	−	±	+	NR	+		NR	
ALS VCP	NR	NR	NR	+	NR	NR	NR	NR	NR		NR	
ALS VAPB	NR	NR	NR	NR	NR	NR	NR	NR	NR		NR	
ALS TDP-43	+	+	−	+	+	+	+	NR	−	RGNEF, Peripherin, pNFH [103]	NR	
ALS FUS	+	+	−	±	+	±	+	+	−	PDI [55] RGNEF, Peripherin, pNFH [103]	PABP1, eIF4G [52]	FUS and ATXN2 [56] FUS and PABP1 [52]
ALS OPTN	+	+	−	+	NR	±	NR	NR	NR		NR	
ALS UBQLN2	+	+	–	+	+	+	+	NR	NR		NR	
ALS ATXN2	+	+	−	NR	NR	NR	NR	+^1^	NR		NR	
ALS C9ORF72	+	+	−	+	NR	+	+	NR	−	DPR^2^ [10, 144] RGNEF, Peripherin, pNFH [103] RBM45 [36]	NR	p62 and DPR [144]

*DPR* dipeptide repeat proteins, *pNFH* phosphorylated high molecular weight neurofilament, *NR* not reported, + aggregates are immunopositive, − aggregates are not immunopositive, ± contradictory results

^1^ATXN2 accumulations are not different between normal and extended repeat carriers

^2^TDP-43 negative, p62, UBQLN, DPR positive inclusions show a specific pattern of distribution in the cerebellum and hippocampus in repeat carriers

Despite clear evidence that protein aggregation is central to the pathology of ALS many questions remain about the role, formation and mechanism-of-action of protein aggregates in ALS. What drives deposition of proteins in ALS? Which cellular mechanisms contribute to protein aggregation or are affected by it? Furthermore, what is the role of proteins carrying ALS-associated mutations in aggregate formation? Pathological, cell culture and animal studies are now beginning to provide insights into these important questions. We will give an overview of the characteristics of aggregates observed in motor neurons of ALS patients and the relationship between protein aggregation, neurotoxicity and disease severity in cellular and animal model systems. We will then discuss possible underlying molecular mechanisms in protein aggregation and neuronal degeneration and provide directions for future research. Although an ever-increasing number of proteins is being implicated in ALS pathogenesis, the emphasis here is on the most recently discovered proteins and those present in spinal cord aggregates.

## Novel insights into the molecular makeup and formation of cellular aggregates in ALS

The central pathological hallmark of ALS is the presence of cytoplasmic inclusions or aggregates in degenerating motor neurons and surrounding oligodendrocytes. Inclusions are not restricted to the spinal cord but also present in other brain regions such as the frontal and temporal cortices, hippocampus and cerebellum [1]. The predominant aggregates found in ALS patients are ubiquitinated aggregates that are classified as either Lewy body-like hyaline inclusions or skein-like inclusions. At the ultrastructural level, Lewy body-like or skein-like inclusions appear as randomly oriented filaments covered by fine granules [78, 129, 166]. Additional subclasses of aggregates found in ALS are Bunina bodies, which are small eosinophilic ubiquitin-negative inclusions [158] and round hyaline inclusions without a halo. Bunina bodies consist of amorphous electron-dense material surrounded by tubular and vesicular structures [158]. Furthermore, neurofilamentous inclusions are found in the axon hillock in close proximity to ubiquitinated inclusions. Other cellular abnormalities include the presence of mitochondrial vacuolization, fragmentation of the Golgi apparatus and abnormalities at the neuromuscular junction. In 1993, SOD1 was the first protein to be identified to aggregate in FALS cases carrying a mutation in the *SOD1* gene [167]. Later, mutations in *VAPB* were also shown to cause ALS in a group of FALS patients [154]. Due to exponential development of genetic techniques, several new proteins have been identified to be involved in ALS pathophysiology during the past few years, including TDP-43, FUS, OPTN, UBQLN2 and C9ORF72. In the following sections, we will discuss for each of these proteins the characteristics of the aggregated protein, their physiological functions and effects in ALS disease models.

### TAR DNA-binding protein 43 (TDP43)

Following the identification of SOD1 aggregates in a small subset of ALS patients, a breakthrough was achieved in 2006 with the identification of TDP-43 as a major component of ubiquitinated inclusions in FTLD and ALS cases [6, 150]. Non-mutated TDP-43 is found in aggregates in spinal cord motor neurons, hippocampal and frontal cortex neurons and glial cells in all SALS patients and the vast majority of SOD-1-negative FALS patients, but not in SOD1 related ALS [133, 181] (Table 1). In frontotemporal dementia (FTD), TDP-43 aggregates are present in the most common subtype of the disease, FTLD with ubiquitinated inclusions, now referred to as FTLD-TDP [150]. ALS and FTD show a remarkable overlap at the genetic, symptomatic and pathological level and they may actually reflect two ends of a disease spectrum [33, 66]. TDP-43 is also found to accumulate in Alzheimer’s disease, Lewy body disease and SCA2, secondary to other molecules [3, 7, 60, 82] and even in normal control subjects over the age of 65 [67].

Normally TDP-43 predominantly localizes to the nucleus. In ALS patients, cytoplasmic aggregation is often accompanied by nuclear clearing of the protein [133, 150]. Furthermore, the protein is cleaved in C-terminal fragments of 18–26 and 35 kDa and the full-length protein and 18–26 kDa fragments are hyperphosphorylated [78, 91, 150]. Genetic studies have identified mutations in the gene encoding TDP-43, *TARDBP,* in 1–2 % of FALS and SALS cases [100, 192]. In non-ALS inclusions TDP-43 is also cleaved and hyperphosphorylated. However, banding patterns of TDP-43 cleavage products are distinct between ALS and FTLD-B and other FTLD subtypes [78, 91, 189]. Furthermore, the distribution of the TDP-43-positive aggregates is disease-specific with, for example, involvement of spinal cord motor neurons in ALS and a more widespread distribution in the brain in FTLD [12]. Mutations in *TARDBP* are unique to ALS and are not found in other neurodegenerative disorders [28, 168, 192] with the exception of a small number of FTD cases [18, 23, 24, 34, 35, 38, 111, 145].

**Fig. 1:**
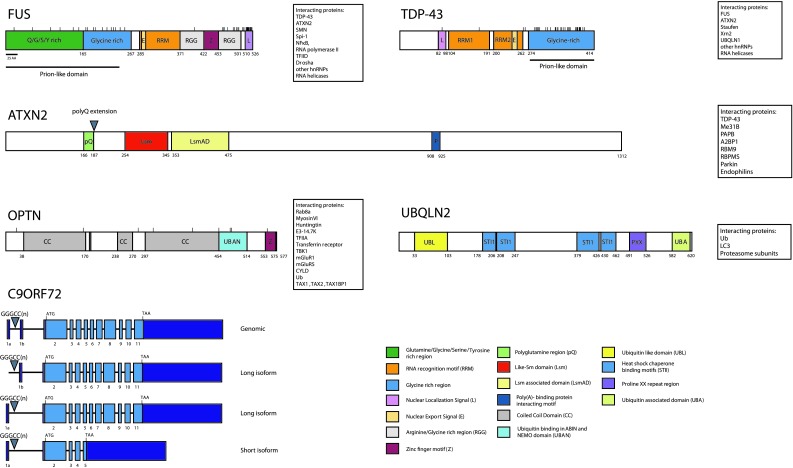
Schematic representation of the domain organization of TDP-43, FUS, ATXN2, OPTN and UBQLN2. Different protein domains are indicated in *different colors* (see legend). The location of ALS-associated genetic alterations is depicted by *sticks* (mutations) or *arrowheads* (repeats). For C9ORF72 intronic and exonic regions are depicted; an intronic hexanucleotide repeat is causative of ALS/FTD (*arrowhead*). *Boxes* show a selection of relevant interactors for each of the depicted proteins

TDP-43 is a DNA and RNA binding protein that binds around 30 % of the mouse transcriptome with a preference for long UG-rich sequences [163, 185] (Fig. 1). Target sequences are mainly intronic, but also include non-coding RNAs and 3′ UTRs. In line with this, TDP-43 plays a role in nuclear RNA metabolism including splicing, transcriptional repression, miRNA synthesis, mRNA nucleo-cytoplasmic shuttling and RNA transport [117]. The majority of TDP-43 mutations identified to date are localized in the C-terminal glycine-rich domain of the protein (Fig. 1). This domain binds other hnRNPs and is important for the splicing activity of TDP-43 [41].

Purified TDP-43 is prone to aggregation and this aggregation tendency depends on its C-terminal domain and is enhanced by ALS-linked TDP-43 mutations [94, 95]. In line with endogenous staining patterns, overexpression of TDP-43 causes a predominant nuclear localization [99, 130, 176, 199]. In addition, a small proportion of primary neuronal cells shows cytoplasmic localization, which is enhanced when TDP-43 mutants are overexpressed [13, 54, 73, 99]. Recently, the first induced pluripotent stem cell (iPSC)-derived motor neurons were generated from fibroblasts of patients carrying a M337V *TARDBP* mutation [22]. Similar to control, the M337V *TARDBP* line showed predominant nuclear localization of TDP-43 but with additional granular staining in soma and neurites [22].

Overexpression of WT TDP-43 has a toxic effect in yeast and cultured primary neurons derived from rat or mouse embryo’s. This effect is even more prominent when mutant TDP-43 is overexpressed [13, 54, 73, 95, 99]. In most animal studies, overexpression of WT or mutant TDP-43 induces a motor phenotype and reduces life span, but results so far are limited to the toxic effects of TDP-43 overexpression [12] (Supplementary Table). Although some studies report on increased toxicity in mutant as compared to WT transgenic lines [9, 180, 222], other studies do not report such differences [177, 212, 213]. As the toxic effects of TDP-43 are clearly dose-dependent [177, 208, 213], some of these results may be dependent on the level of expression rather than on TDP-43 mutation-specific toxic effects. Furthermore, while it has been reported that TDP-43 WT overexpression has motor neuron-specific toxic effects coinciding with the presence of nuclear and cytoplasmic inclusions and pathological phosphorylation and cleavage of TDP-43 [89, 208, 213], overexpression of TDP-43 can be toxic without aggregate formation [9, 205].

Several factors modulate TDP-43 toxicity. First, the TDP-43 C-terminal fragments that are found in ALS spinal cord aggregates are particularly aggregation-prone and toxic when overexpressed [13, 54, 88, 156, 221]. Second, the cytoplasmic distribution of TDP-43 has been reported to be related to cell death whereas inclusion body formation or nuclear TDP-43 levels were not [13]. In line with this, mutations of the nuclear localization signal (NLS) of TDP-43 confer toxicity while NES mutations abrogate toxicity [13]. Third, phosphorylation of TDP-43 may modulate aggregation and toxicity of the protein [26, 126]. In *C. elegans* blockage of TDP-43 phosphorylation ameliorates the neurodegenerative effect of ALS-associated TDP-43 mutants [126, 127]. In contrast, another study reported that mutation of phosphorylation sites increased aggregate formation in cells and in *Drosophila*, whereas hyperphosphorylation reduced aggregation and toxicity [123]. In addition, mutation of TDP-43 phosphorylation sites does not affect C-terminal fragment formation, the formation of cytoplasmic inclusions or survival in cells [51, 153, 221]. Thus, although it is clear that TDP-43 plays a crucial and central role in ALS pathology many questions remain about its pathological mechanisms in ALS.

### Fused in sarcoma/translocated in liposarcoma (FUS/TLS)

The discovery of ALS-linked mutations in TDP-43 fueled the identification of mutations in another RNA binding protein, *FUS,* in FALS patients [116, 196]. A series of genetic studies of large ALS cohorts showed that *FUS* mutations account for 4 % of FALS and 1 % of SALS cases and that these are in part associated with young-onset disease [117]. Pathological examination of post-mortem tissue of FUS mutation carriers shows predominant degeneration of lower motor neurons with FUS-positive cytoplasmic inclusions and a normal distribution of TDP-43, thereby distinguishing them from other ALS cases [72, 80, 116, 196]. The precise pattern of FUS-immunoreactivity in ALS cases without FUS mutations is still unclear (Table 1). Some studies report that FUS is not present in SALS patients and SOD1 FALS [87, 149, 196], while others show FUS-positive inclusions with signals for TDP-43, p62 and ubiquitin in all SALS and FALS cases, except for SOD1 mutation carriers [48, 103]. In contrast to TDP-43, biochemical analyses show that mutant FUS protein itself is not ubiquitinated, hyperphosphorylated or cleaved. However, the protein is enriched in the insoluble fraction of FTLD-FUS brains [149]. Furthermore, nuclear clearing of FUS is not as evident as observed for TDP-43.

FUS has also been detected in aggregates found in other neurodegenerative disorders including FTLD, Huntington’s disease and SCA indicating that disruption of WT FUS function is associated with neurodegenerative disease in general [50, 149]. However, in contrast to ALS, FTLD patients with FUS inclusions only rarely harbor genetic alterations in *FUS* [195]. Interestingly, FUS-positive inclusions in FTLD cases are immunoreactive for TAF15 and EWS, other members of the FET family of RNA binding proteins, and for transportin-1 [147, 151]. Protein aggregates in ALS cases with FUS mutations do not stain for these proteins.

FUS is a nuclear RNA binding protein and preferentially binds pre-mRNA at intronic sites, but also long non-coding RNAs, exons and 3′UTRs [84] (Fig. 1). Most of the ALS-linked FUS mutations reported to date are localized in the NLS of the protein resulting in impaired nuclear transport of FUS [52]. FUS mediates a wide range of cellular processes including DNA repair, transcription, splicing and miRNA processing [117]. The protein shuttles between the nucleus and the cytoplasm to function in the transport of mRNA [224].

Purified FUS is aggregation prone, but in contrast to TDP-43 this property may not be influenced by ALS-linked mutations [179]. In yeast, cytosolic aggregation of FUS depends on its N-terminal, RRM and first RGG domains (Fig. 1). Toxicity is dependent on the N-terminal and first RGG domains [97, 179]. It should be noted, however, that the NLS of FUS is not fully recognized in yeast suggesting that this domain may still be involved in protein aggregation. Following overexpression in eukaryotic cells, WT FUS localizes to the nucleus, whereas FUS proteins carrying ALS mutations in the NLS form cytoplasmic aggregates, thereby mimicking human disease [116, 196]. This cytoplasmic relocalization is observed in some but not all FUS animal models reported to date [25, 32, 42, 86, 119, 142, 198, 203, 211] (Supplementary Table).

Deletion of the NES in FUS strongly reduces toxicity of mutant FUS in *Drosophila* [119], suggesting that the cytoplasmic localization of mutant FUS confers toxicity. However, another study reported that deletion of the NLS completely blocks FUS toxicity, as did the addition of a NES [211]. Blocking the RNA binding capacity of FUS also abolishes FUS toxicity in yeast [178] and ameliorates mutant FUS toxicity in *Drosophila* [42] (Supplementary Table). Although further work is needed to reveal to which extent mislocalization of mutant FUS contributes to disease, recent studies show that mutant FUS triggers stress granule formation and loss of nuclear GEMs, as will be discussed in more detail in the next section.

### Optineurin (OPTN)

A study on Japanese ALS patients from consanguineous marriages reported mutations in *OPTN* to be associated with disease [135]. Although genetic variation in OPTN is rare in ALS patients in other populations, pathologic studies confirm a role for OPTN in ALS. In SALS cases, OPTN is present in cytoplasmic skein-like inclusions and colocalizes with ubiquitin, TDP-43, and possibly FUS [46, 85, 103, 135, 159] (Table 1). Conflicting results are obtained with respect to OPTN immunoreactivity in SOD1 and FUS mutation carriers. Two studies detected colocalization of OPTN with SOD1-positive inclusions in SOD1 mutation carriers [103, 135], while two other studies did not [46, 85]. Similarly, two studies reported OPTN immunoreactivity in FUS mutation carriers [92, 103], while a second group could not detect OPTN-positive inclusions [85].

OPTN is present in inclusions in several other neurodegenerative diseases such as ALS with dementia, Huntington’s disease, Alzheimer’s disease, Parkinson’s disease, Creutzfeldt-Jakob disease, multiple system atrophy and Pick disease [159].

OPTN functions as an inhibitor of NFκB-signaling [223], acts as an autophagy receptor [206] and participates in the regulation of vesicular trafficking and maintenance of the Golgi apparatus [169] (Fig. 1). Purified OPTN is not aggregation prone although strong overexpression in yeast results in aggregation of the protein and in toxicity [113]. This toxicity requires the Rab8 binding region but not the ubiquitin-binding domain of OPTN. Mutations identified in ALS patients so far include truncation mutations thought to act through loss-of-function mechanisms and missense mutations [17, 45, 90, 135, 141, 191]. OPTN E478G, carrying a mutation in the UBAN domain, looses its ability to bind K63-polyubiquitin or linear-polyubiquitin chains [206] and fails to inhibit NFκB [135]. Whereas exogenous WT OPTN localizes to LC3-positive vesicles upon autophagy induction [174, 206], OPTN E478G does not [206]. As the ubiquitin binding capacity of OPTN is necessary to serve as an LC3 adaptor, this likely reflects a loss of binding to autophagosomes [206]. However, whether the inability of OPTN E478G to bind ubiquitin is related to ALS pathophysiology is unknown. It is interesting to note that homozygous knock-in mice expressing a OPTN D477N mutant, which also lacks ubiquitin binding capacity, do not display an ALS-like phenotype [70]. The OPTN truncation mutants reported in ALS patients have been proposed to cause decreased OPTN protein levels. In this light it is interesting that knockdown of OPTN results in motor neuron phenotypes in zebrafish [110]. Whether incorporation of OPTN in ALS aggregates in non-mutation carriers also results in loss of function of the protein or merely reflects its role as an autophagic receptor warrants further investigation.

### Ubiquilin-2 (UBQLN2)

Dysfunction of the ubiquitin–proteasome system (UPS) has been linked to ALS based on a variety of functional studies. The contribution of this process to motor neuron degeneration is further underlined by the recent identification of mutations in *UBQLN2* in X-linked ALS/FTD [47]. In human spinal cord autopsy material of UBQLN2 mutation carriers, skein-like inclusions are positive for UBQLN2, ubiquitin, p62, TDP-43, FUS and OPTN but not SOD1 [47, 207] (Table 1). In cases with ALS-dementia with or without UBQLN2 mutations, UBQLN2-positive inclusions are found in the hippocampus which are absent in ALS cases without dementia indicating that UBQLN2 aggregation and neurodegeneration are linked [47]. Skein-like inclusions in spinal cord tissue from SALS and FALS patients with unknown mutations or mutations in SOD1, TDP-43 or FUS also stain positive for UBQLN2 [47, 207]. It is currently unknown whether UBQLN2 is present in aggregates in other neurodegenerative diseases and a first study did not detect mutations in UBQLN2 in FTD [79]. Whether the presence of UBQLN2 in ALS aggregates reflects a cellular attempt for protein degradation or is related to dysfunction of protein degradation pathways needs to be further investigated.

The exact function of UBQLN2 is unknown, but it has been implicated in protein degradation via both UPS and autophagy and in G-protein coupled receptor endocytosis [121] (Fig. 1). Overexpression of mutant UBQLN2 has been shown to result in impaired UPS function [47]. Most, but not all ALS-associated mutations identified in UBQLN2 to date involve proline residues in its PXX region, which is thought to be important for protein–protein-interactions. Further research is needed to determine how mutant UBQLN2 impairs protein degradation systems and which proteins are affected.

### Ataxin-2 (ATXN2)

A yeast screen for modifiers of TDP-43 toxicity recently led to the discovery that extended polyQ repeats in *ATXN2* are associated with ALS [53]. While ATXN2 normally harbors 21 or 22 polyQ repeats, and a polyQ length of 34 and higher is known to cause SCA2 [120], polyQ lengths between 27 and 33 are associated with ALS [53]. Spinal cord tissue of SALS patients shows an increased cytoplasmic accumulation of ATXN2, as compared to controls, but there is no difference between patients with normal or extended polyQ repeats [53] (Table 1). Furthermore, ATXN2 and TDP-43 colocalize in cytoplasmic inclusions in FTLD [53], and FUS and ATXN2 have been reported to colocalize in ALS [56].

ATXN2 functions in mRNA polyadenylation, stress granule formation, polyribosome assembly and miRNA synthesis [136, 157, 171] (Fig. 1). The pathological effect of extended ATXN2 polyQ repeats is likely due to a gain-of-function mechanism as *ATXN2* knockout mice do not show overt neurological deficits [104]. In contrast to SCA2, the polyQ repeats associated with ALS are not pure, i.e., containing only CAG codons. Rather, they are composed of CAG codons interrupted by CAA codons [216]. As both codons encode for the same amino acid, the pathogenic effect of ATXN2 polyQ repeat extensions may reside at the mRNA level.

Overexpression of ATXN2 with intermediate length polyQ repeats does not affect ATXN2 localization [53]. However, there is evidence that subcellular distribution of overexpressed WT TDP-43 or mutant FUS is altered upon overexpression of ATXN2 with intermediate length polyQ repeats [53, 56, 152]. Furthermore, ATXN2 with intermediate length polyQ repeats enhances stress-induced activation of caspase-3 as well as cleavage and phosphorylation of TDP-43 [77]. So, there is evidence that ATXN2 intermediate polyQ repeat modulates ALS pathophysiology via its RNA-dependent interaction with FUS and TDP-43, but further research is needed to dissect the underlying molecular mechanisms.

### C9ORF72

An intronic hexanucleotide repeat expansion in *C9ORF72* was recently identified as the most prevalent cause of ALS, FTD and ALS-FTD [44, 164]. While in the wild type situation the gene harbors fewer than 25 repeats, the repeat region can be extended to several hundred or thousand repeats [15]. Although extended repeat lengths can also be detected in control cases, repeat expansions are strongly associated with ALS and FTD. Extended repeat lengths have also been reported in some cases of Alzheimer’s disease and Huntington disease-like syndrome [15, 134, 209]. C9ORF72 is a protein with unknown function, but shows homology to differentially expressed in normal and neoplasia (DENN), which is a GDP/GTP exchange factor (GEF) that activates Rab GTPases [122, 217] (Fig. 1). The expression pattern of C9ORF72 is unaltered in expanded repeat carriers, although the specificity of the available C9ORF72 antibodies is subject to debate (Table 1). TDP-43-negative, p62- and UBQLN-positive cytoplasmic and nuclear inclusions in the hippocampus, frontotemporal neocortex and cerebellum distinguish expanded repeat from non-expanded repeat carriers [2, 27]. Fascinatingly, these characteristic inclusions contain poly dipeptide repeat proteins generated by non-ATG-initiated translation from the C9ORF72 intronic repeat region [10, 144]. Whether and how these dipeptide repeat proteins mediate pathogenic effects are unknown and they probably represent one of several pathogenic mechanisms in C9ORF72-associated ALS. As a second mechanism, *C9ORF72* RNA molecules containing extended repeats may accumulate and sequester RNA binding proteins preventing these proteins from exerting their crucial functions. Similar aggregation of mutant RNAs is observed in other repeat expansion disorders. In support of this, *C9ORF72*-containing RNA foci have been observed in 25 % of spinal and frontal cortical neurons of expanded repeat carriers compared to 1 % in controls [44]. This observation has, however, not been confirmed in a second, independent study [175]. It has been shown that the GGGGCC repeats present in C9ORF72 bind several RNA binding proteins and that one of these, hnRNPA3, localizes to the p62-positive/TDP-43-negative cytoplasmic inclusions observed in repeat carriers [143]. As a third mechanism, the repeat expansion may result in haploinsufficiency due to impaired transcription or splicing. This is supported by the finding that C9ORF72 protein levels are reduced in patients with increased repeat lengths [44, 68, 144, 193]. Finally, in a number of C9ORF72 repeat carriers with FTLD, tau pathology has been observed suggesting that the C9ORF72 repeat may influence tau protein [20, 106]. Further research is needed to address these different mechanisms in relation to ALS disease pathogenesis.

## Molecular mechanisms underlying protein aggregation in ALS

When studying ALS pathophysiology it is essential, but very difficult, to distinguish cause and consequence in the cellular cascades driving protein aggregation. The recent discovery of new, disease-associated mutations that trigger protein aggregation or stability represent unique opportunities to further dissect the effect and mechanism-of-action of protein aggregation in ALS. Several important questions need to be addressed including (1) how are proteins sequestered into ALS aggregates and (2) how do these aggregates affect neuronal function? Several models addressing these questions have emerged and will be discussed below.

### Low complexity domains in ALS proteins with aggregation-prone properties

**Fig. 2:**
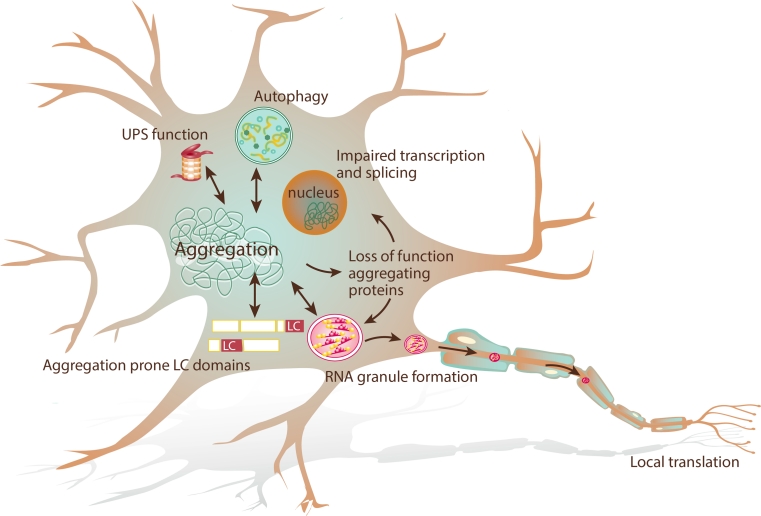
Cellular mechanisms linked to protein aggregation in ALS. ALS-associated mutations result in cytoplasmic mislocalization or increased aggregation tendency thereby increasing the risk for aberrant aggregation. Proteins with domains of low complexity (prion-like domains) such as FUS, TDP-43 and HNRNPA1 are though to be intrinsically aggregation prone. Many of these proteins participate in RNA granule formation. ALS-associated mutants alter RNA granule formation, thereby interfering with the local translation of RNA. Moreover, sequestration of ALS-associated proteins and their interactors into cytoplasmic aggregates may result in a loss of function. Protein degradation by the UPS and autophagy is essential for the clearance of ubiquitinated proteins. Dysfunction of these systems, as has been suggested for mutant UBQLN2, OPTN and VCP, can lead to proteins deposits

FUS, TDP-43 and other RNA binding proteins (RBPs) contain domains with similarity to yeast prion domains (Figs. 1, 2). These domains are enriched for asparagine, glutamine, tyrosine and glycine residues and can adopt two conformational states: an unfolded and an aggregated state. Prion proteins in an aggregated state can sequester prion proteins in an unfolded state to adopt the aggregation-prone confirmation and as such aggregation can spread. It has been hypothesized that in ALS aggregation may propagate from one cell to the other in a comparable fashion [107, 162]. Aggregation of FUS and TDP-43 has been shown to rely on regions resembling prion domains [94, 102, 179] and mutations in TDP-43 associated with ALS occur mainly in its prion-like region (Fig. 1). Furthermore, there is direct evidence that ALS proteins share features with yeast prion proteins, e.g., that aggregated FUS or TDP-43 can sequester native protein [62, 64, 102, 215]. Finally, mutations in the prion-like domain of the RBP hnRNPA1 segregate with disease in FALS [105]. Yeast prions form amyloid deposits characterized by a secondary beta-sheet structure and stain positive for amyloid dyes. Although short synthetic TDP-43 and FUS peptides form amyloid-like fibrils in vitro [31, 73, 102] full-length TDP-43 does not [95]. Rather, purified full-length FUS and TDP-43 form pore-like oligomers and fibrils resembling the ultrastructure of the skein-like inclusions [95, 179]. However, although initial reports emphasized that ALS inclusions lack features of amyloid [148], two recent studies found that some TDP-43 positive inclusions stain positive for amyloid dyes [21, 166]. Furthermore, to date evidence for cell-to-cell spread of ALS aggregated proteins is lacking. Finally, toxicity induced by TDP-43 and FUS depends not only on prion-like domains but also on RNA binding properties [42, 53, 94, 179, 199]. In all, accumulating evidence hints at an important role for prion-like mechanisms in ALS pathogenesis but further studies are needed to uncover their precise mechanism-of-action and pathological effects.

### Stress granule formation and ALS protein aggregation

Although FUS and TDP-43 have a predominant nuclear localization, both proteins rapidly shuttle between the nucleus and the cytoplasm and are actively transported into axons and colocalize with other RBPs [16, 54, 63, 186, 202, 224]. RBPs regulate local translation of mRNAs by forming granular RNA–protein complexes in which translation is repressed [5]. Several types of RNA granules can be distinguished: stress granules (SG), processing bodies (PBs) and so called neuronal or transport granules. These granules are highly dynamic structures and interaction and exchange of RBPs and transcripts occur between the different types of granules [5]. SGs or PBs are formed following polysome disassembly in response to stress, while neuronal granules serve in the transport of mRNA to their site of local translation [5]. Interestingly, it has been suggested that the aggregation-prone prion-like domains of RBPs assist in the dynamic movement of RBPs in and out of RNA granules [102].

ALS-associated mutations in TDP-43, FUS and ATXN2 are being linked to SGs (Fig. 2). SG formation depends on the RRM domains and C-terminal region of TDP-43, the same domains required for TDP-43 toxicity [19, 37]. Although TDP-43 is dispensable for SG formation [19, 37, 131], depletion of TDP-43 inhibits SG formation [137]. WT TDP-43 co-immunoprecipitates and colocalizes with stress granule markers, but the effect of mutant TDP-43 is less clear [37, 43, 49, 61, 83, 131, 137, 138, 160]. While two studies reported an increase in the number of cells containing SGs upon TDP-43 mutant overexpression [49, 131], another study failed to observe such an effect [137]. A fourth study did not detect cytoplasmic signals for WT or mutant TDP-43 and consequently no colocalization with SG markers, although a TDP-43 NLS mutant clearly localized to SGs [19]. Thus, WT TDP-43 likely plays a role in SG formation with some evidence for altered SG formation associated with mutant TDP-43.

In contrast to TDP-43, mutant but not WT FUS localizes to SGs [19, 25, 52, 108, 152, 197]. Whether mutant FUS induces SG formation or is recruited to SGs following stress is unclear [108, 152, 197]. Similarly, studies reporting the effect of FUS mutations or truncations on SG recruitment show conflicting results [19, 102, 108]. However, proteins involved in SG formation have been identified as modifiers of FUS toxicity in yeast screens [97, 179].

ATXN2 localizes to PBs and SGs following cellular stress and its Lsm domain and PAM2 motif are required for this localization [152, 157] (Fig. 1). It is unknown whether ATXN2 directly regulates RNA granule formation or function. However, overexpression of ATXN2 31Q increases the cytoplasmic localization of TDP-43 following heat shock [53] and increases the cytoplasmic localization of mutant FUS, but without affecting SG formation [56].

In conclusion, FUS, TDP-43 and ATXN2 have all been implicated in SG formation and there is evidence that alterations in SG formation are associated with ALS-associated mutant proteins. Interestingly, HNRNPA1 localizes to SGs as well and ALS-associated mutations in HNRNPA1 augment its incorporation into SGs [105]. However, while the formation of RNA granules is normally a reversible process, in ALS disrupted RNA granule formation is hypothesized to result in insoluble aggregates. The most convincing evidence for this comes from pathological examination of ALS spinal cords. Although one study did not detect colocalization of TDP-43 positive inclusions with SG markers in SALS motor neurons [37], three other studies, using more sensitive methods, convincingly did [19, 131, 200]. Furthermore, the SG markers PABP-1 and eIF4G colocalize with FUS aggregates in spinal cord of FUS mutation carriers [52]. Whether these aggregates have primarily formed as SGs or reflect a more general sequestration of interacting proteins in FUS and TDP-43 aggregates (see below), remains to be shown. Furthermore, how reversible SGs develop into insoluble aggregates is unknown. Increased cytoplasmic localization of FUS and TDP-43, increased aggregation tendency of mutant RBPs and phosphorylation of RBPs have been suggested to regulate their localization to SGs [102, 108, 138] and may be altered in ALS. An altered RNA binding preference of mutant RBPs could also underlie altered RNA granule formation [84]. How RNA granule formation and disassembly are dysregulated in ALS and which transcripts are affected by these processes will undoubtedly be a focus of further investigations.

### Protein sequestering

Cytoplasmic aggregation of ALS proteins at ectopic sites in the cell may prevent these proteins from executing their normal function (Fig. 2). If this mechanism would be solely responsible for ALS pathogenesis, gene knockdown or knockout is expected to result in strong motor neuron phenotypes. Unfortunately, FUS and TDP-43 are essential for normal development and knockout of FUS or TDP-43 results in premature death in mice [81, 112, 115, 172]. There is some evidence that knockdown of FUS or TDP-43 results in motor dysfunction in *Drosophila* and zebrafish models [58, 98, 99, 128, 170, 203] (Supplementary Table). However, the relevance of these findings in the context ALS is unclear as knockdown often triggers abnormalities outside the nervous system as well and motor neuron degeneration is not consistently observed. Additional models are needed to study the effect of reduced levels of proteins like FUS and TDP-43 but it seems unlikely that reduced levels of these proteins alone cause ALS.

On the other hand, FUS and TDP-43 aggregation could exert a toxic effect via sequestration of multiple binding partners or even interactomes essential for neuronal function. FUS and TDP-43 interact and colocalize with many different proteins including SMN, gemin proteins and small nuclear ribonucleoprotein particles (snRNPs) [188, 214] (Fig. 1). The SMN complex can be detected in the cytoplasm, but also in nuclear foci called GEMs. Knockdown of FUS or TDP-43 results in the loss of GEMs [173, 188]. Furthermore, a reduced number of GEMs is observed in ALS spinal cord motor neurons [188], fibroblasts derived from FUS or TDP-43 mutation carriers [214] and ALS mouse models [65, 101, 173]. Therefore, sequestration of SMN in FUS or TDP-43 cytoplasmic aggregates could affect SMN levels and function.

The idea that aggregation of proteins, such as TDP-43, leads to the sequestering of other essential proteins is supported by a recent study identifying the lariat debranching enzyme Dbr1 as a modifier of TDP-43 toxicity [8]. Dbr1 normally mediates the degradation of intronic lariats. Knockdown of Dbr1 triggers the transport of excessive lariat RNAs into the cytoplasm where they bind TDP-43. Since this leads to a reduction in cellular toxicity it was proposed that knockdown of Dbr1 reduces TDP-43 toxicity by capturing TDP-43 and thereby diminishing sequestration of RNAs and RBPs in TDP-43 aggregates. Although currently functional evidence for RNA and protein sequestering by ALS-associated protein aggregates is scarce, these recent findings warrant a more extensive study of the ability of ALS-associated protein aggregates to sequester RNAs and RBPs and of the effect this has on motor neuron homeostasis.

### Dysfunction of protein degradation pathways

Molecular chaperones, the UPS, and the autophagy-lysosome system function to monitor protein quality and protect cells from dysfunctional, malfolded or denatured proteins. The presence of ubiquitin, p62 and molecular chaperones in ALS aggregates implicates a role for all these three systems in ALS pathophysiology (Fig. 2).

Chaperone molecules assist in protein folding under physiological circumstances and prevent protein aggregation in response to stress. They also assist in protein degradation by the proteasome or autophagy. Chaperones, such as heat shock proteins, are upregulated in ALS spinal cord [4] and present in motor neuron aggregates [14, 204]. Interestingly, upregulation of molecular chaperones increases solubility and reduces toxicity of FUS [139] and TDP-43, especially of TDP-43 C-terminal fragments [40, 71]. In line with this, knockdown of molecular chaperones increases accumulation of pTDP-43 C-terminal fragments [220] and enhances toxicity of TDP-43 overexpression [219]. Thus, chaperone molecules are likely to play a significant role in aggregation of ALS proteins and in their toxic effects.

Ubiquitination not only marks proteins for degradation but also mediates intracellular signaling, e.g., NFκB activation [114]. As proteins present in ALS aggregates are ubiquitinated it has been postulated that they are marked for degradation by the UPS, but eventually deposit when dysfunctional protein levels exceed UPS capacity. In line with this, examination of ALS spinal cord tissue shows that ubiquitination occurs before accumulation starts and that inclusion formation inversely correlates to the number of motor neurons, indicating that deposition of ubiquitinated proteins relates to toxicity [69]. Consistently, proteasome inhibition has been found to increase endogenous TDP-43 levels, blocks degradation of overexpressed TDP-43 C-terminal fragments and enhances toxicity [88, 155, 194, 220]. Recently, motor neuron-specific knockout of the proteasome subunit Rpt3 was reported to result in the loss of spinal motor neurons and locomotor dysfunction in mice [183]. Interestingly, these mice contained basophilic, hyaline and skein-like inclusions positive for TDP-43, FUS, UBQLN2 and OPTN, indicating that dysfunction of the proteasome itself is sufficient to induce aggregation of ALS-associated proteins and motor neuron degeneration. The fact that mutations in UBQLN2 cause ALS and are associated with impaired UPS function further underlines the notion that proteasomal degradation does not only modulate but also may play a causative role in ALS pathogenesis.

p62 serves as an adaptor of autophagic degradation by binding both polyubiquitinated proteins and LC3, an important autophagosomal marker. Autophagy mediates the degradation of TDP-43 C-terminal fragments, but not full-length protein [30, 93, 182]. Interestingly, treatment of transgenic TDP-43 mice with autophagic activators reduces locomotor dysfunction, learning and memory deficits, and neuron loss [201]. This is accompanied by a decrease in cytosolic TDP-43 inclusions and in insoluble full-length and truncated TDP-43. This indicates that autophagic clearance of TDP-43 reduces neurotoxicity. Knockout mice lacking Atg7 or Atg5 in the central nervous system, genes essential for autophagy, display movement disorders, widespread neurodegeneration and ubiquitin-positive inclusions in a variety of brain regions [75, 109]. Motor neuron-specific knockout of these genes results in ubiquitinated and p62-positive inclusions, but do not stain for TDP-43, FUS, OPTN or UBQLN2 and do not result in motor neuron death [183]. This would suggest that autophagic disruption does not primarily underlie ALS pathogenesis. However, mutations in genes encoding autophagy regulators have been associated with ALS, i.e., VCP, p62, CHMP2B and UBQLN2 [47, 57, 96, 161]. Patients with mutations in *SQSTM1*, the gene encoding p62, show large round p62-positive inclusions in motor neurons with additional TDP-43 deposits which are p62-negative [184]. Furthermore, TDP-43 has been identified as a modifier of mutant VCP toxicity [165] and ubiquilins have been reported to modulate TDP-43 toxicity in *Drosophila* [74]. In conclusion, components of the protein degradation pathways have emerged as important modulators of protein aggregation and toxicity in ALS. Furthermore, mutations in genes encoding these components have been associated with ALS indicating a causal role in the disease. Future studies are needed to investigate how these processes are affected in ALS, as they may represent potential therapeutic targets.

## Conclusions

Protein aggregation in affected motor neurons is a central hallmark of ALS, and recent genetic, cellular and histological studies have enlarged our understanding of the molecular composition of these aggregates. This has led to the identification of new ALS causing genes, has linked the composition of aggregates to specific genetic defects, and has provided starting points for further investigation of underlying molecular pathways. Unique pathological features have now been identified that distinguish FUS, SOD1 and C9ORF72 mutation carriers from other ALS cases. It is likely that with future discoveries further classification will be possible. TDP-43, FUS, p62, OPTN and UBQLN2 show a widespread distribution in ALS-linked aggregates. This could reflect a general role in pathogenesis or, on the contrary, question their specific relevance to disease. However, the fact that mutations in the genes encoding these proteins segregate with disease in FALS supports the idea that their dysfunction is linked to motor neuron degeneration and disease pathogenesis. The proteins identified to be present in ALS aggregates play a role in a wide range of cellular processes with a marked overlapping role for TDP-43, FUS and ATXN2 in RNA metabolism and for OPTN, UBQLN2 and VCP in protein quality control and degradation (Fig. 2). How a disturbance of these ubiquitously expressed proteins can result in motor-neuron-specific degeneration remains an unresolved issue in the field of ALS research. Although cellular and animal models confirm a role for aggregation in ALS, results are often contradictory and models fully recapitulating ALS pathogenesis are mostly lacking. This may in part be explained by the fact that many of these models rely on overexpression of the protein. New model systems, such as iPSC-generated patient-derived cell lines or inducible animal models, may help to overcome these problems. So far, TDP-43 and FUS have been investigated most extensively; future studies on the role of OPTN, UBQLN2, ATXN2 and C9ORF72 will further enlarge our understanding of the cellular processes underlying ALS. Processes underlying aggregation in ALS include enhanced intrinsic aggregation propensity of ALS proteins, RNA granule dysregulation and dysfunction of protein degradation pathways. A further understanding of these processes will not only deepen our understanding of ALS pathogenesis, but also may aid the development of novel therapeutic strategies for this disease.

## Electronic supplementary material

Below is the link to the electronic supplementary material.

Supplementary material (DOCX 136 kb)
