# Supplementary material for: Protein aggregation in amyotrophic lateral sclerosis
Source: Acta Neuropathol. 2013 May 15;125(6):777–94. doi: 10.1007/s00401-013-1125-6 (PMC3661910; doi:10.1007/s00401-013-1125-6)
Supplement: Supplementary file 1 — Supplementary material (DOCX 136 kb) [file 401_2013_1125_MOESM1_ESM.docx]

**Table**

| **Model system** | **Promotor** | **Construct** | **Protein localization** | **Aggregation/**  **Insolubility** | **Life span** | **Phenotype** | **Neuronal death/defects** | **Reference** |
| --- | --- | --- | --- | --- | --- | --- | --- | --- |
| **TDP-43** |  |  |  |  |  |  |  |  |
| *C. elegans* | Snb-1  (pan-neuronal) | hTDP-43 WT  M337V  A315T  G290A | Nucleus | WT and mutants: NIIs TDP-43+, HP, CTF | Reduced  WT = mutants | Motor deficits  Mutant > WT | Degeneration of GABA-ergic neurons in mutants not WT | [126] |
| *C. elegans* | Snb-1 | hTDP-43 WT  TDP-1  dRRM1  dRRM2  dC-terminus  CTF | Nucleus  CTF: cytoplasm | No, except CTF | NR | hTDP-43/TDP-1: uncoordinated phenotype  Deletion of RRM1, RRM2 or C-terminus: no phenotype | No GABA-ergic motor neurons loss  Abnormal synapses | [11] |
| *C. elegans* | Snb-1 | hTDP43 WT  Q331K  M337V  CTF | WT, Q331K, M337V: nucleus, CTF: cytoplasm | CTF: NCIs TDP-43+  WT and mutants: no visible cytoplasmic aggregation  TDP-43 present in insoluble fraction | NR | Reduced motility Mutant=WT | No | [220] |
| *C. elegans* | Unc-47 (GABA-ergic motor neurons) | hTDP-43 WT  A315T | A315T: nucleus and cytoplasm  WT NR | TDP-43 A315T in insoluble fraction more than WT | Normal | Progressive paralysis  Mutant > WT | Degeneration of motor neurons  Mutant>WT | [190] |
| *C. elegans* | NA | TDP-1 deletion | NR | NA | NR | No movement disorders | No synaptic abnormalities in GABA-ergic neurons | [11] |
| *C. elegans* | NA | Deletion of 1.2 kb of tdp-1 (including RRM1, RRM2 and glycine-rich domain) | NA | NR | Increased | Deficits in fertility, growth and locomotion | NR | [218] |
| *C. elegans* | NA | Knockdown | NR | NA | Increased | NR | NR | [190] |
| *Drosophila* | 221 (sensory neurons) | HTDP-43 WT  M337V  Q331K | NR | NR | NR | NR | Increased dendritic branching  WT>mutants | [132] |
| *Drosophila* | Actin5C (ubiquitous, strong)  Elav4 (pan-neuronal)  D42 (motor neurons) | hTDP-43 WT  dNLS  FFLL (loss of RNA binding capacity)  G287S  A315T  G348C  A382T  N390D  CTF | All nucleus except dNLS and CTF | NR | Actin: all lethal except FFLL and CTF  Elav:  Reduced life span WT>mutants | D42: reduced climbing ability  WT>A315T>FFLL>CTF | NR | [199] |
| *Drosophila* | GMR (eye)  OK107 (mushroom bodies)  OK 371 (motor neurons)  RN2 (small subset of motor neurons) | hTDP-43 WT | OK371: WT mainly nuclear, some cytoplasmic aggregation  OK107 and GMR: NR | OK371: NCIs TDP-43+  GMR: TDP-43 in insoluble fraction | OK 371: reduced | GMR: WT eye phenotype  OK371: WT failure to hatch  RN2: WT Motor defects | OK107: WT neuronal and axonal loss  OK371: WT cell death of motor neurons | [124] |
| *Drosophila* | GMR | hTDP-43 WT  M337V  dNLS  dNES | WT and dNES: nucleus  dNLS: cytoplasmic | NR | NR | Rough eye phenotype  dNLS>M337V>WT>dNES | Degeneration of photoreceptors | [165] |
| *Drosophila* | GMR  D42 | hTDP-43 WT | Nucleus | No | D42: reduced | D42: Movement defects, paralysis | GMR: depigmentation of the eye  D42: motor neuron loss | [74] |
| *Drosophila* | Elav  D42  OK107  MHC (muscle) | dTDP | WT: nucleus and cytoplasm | NCIs TDP-43+ | Dose-dependent reduction in survival | Reduced larval and adult locomotor activities. | Increased bouton number and axon branches at NMJ | [128] |
| *Drosophila* | GMR  24B (muscle)  Repo (glia)  Elav-inducible | hTDP-43 WT  dNLS  dNES | GMR – WT and dNLS: nucleus and cytoplasm  Elav-ind: nucleus except dNLS | Elav-ind: Nuclear aggregation, CTF | Elav, 24B, Repo: all lethal.  Elav-ind: reduced WT>dNLS>dNES | GMR: Eye phenotype dNLS>WT>NES  ElavGal4 24B: normal | NR | [140] |
| *Drosophila* | Actin (whole-body)  OK371 | hTDP-43 WT  A315T | NR | NR | Actin: lethal  OK371: WT and mutant reduced | OK 371: movement disorders  Mutant>WT>control | OK 371: WT and mutant: axonal and neuronal loss, axonal swelling | [73] |
| *Drosophila* | NA | TBPH knock-out chromosomal deletion  Knockdown using RNAi | NR | NR | Reduced | Knockout: entrapped in pupal cages, spastic uncoordinated movements, incapacity to fly  RNAi shows a comparable phenotype | NMJ defecits: reduced number of axonal branches and synaptic boutons | [58] |
| *Drosophila* | NA | Truncation mutant  RNAi | NR | NR | Homozygous truncation mutant: semi-lethal | NR | Decreased dendritic branching | [132] |
| *Drosophila* | NA | Deletion of complete coding region | NR | NR | Died as second instar larvae | NA | NA | [59] |
| *Drosophila* | NA | Knockdown  RNAi | NR | NR | NR | No eye phenotype  No locomotion defects | Axon loss and neuronal death in mushroom bodies | [124] |
| *Drosophila* | D42  Elav  OK107  MHC | Knockdown GAL4 system  Knockout via P-element excision | NR | NR | OK107: normal  Knockout: semi-lethal | Knockdown D42 and MHC: no effect  Elav: locomotion defects  Knockout: Locomotion defects | Increase in number of boutons at NMJ | [128] |
| *Drosophila* | Actin | Knockdown | NR | NR | NR | Increased production of sensory bristles and sensory organ precursor (SOP) cells | NR | [125] |
| *Mouse* | Prp (ubiquitous) | A315T | Nucleus | NCIs Ub+ TDP-43-  CTF i | Reduced | Gait abnormalities  Weight loss | Widespread motor neuron degeneration | [205] |
| *Mouse* | Thy-1 (neurons and glia) | hTDP-43 WT | Nucleus | NCIs and NIIs  NII TDP-43+, NCIs partly TDP-43+  CTF | Reduced | Gait abnormalities, paralysis, fasciculation, spasms  Weight loss | Loss of spinal and cortical motor neurons | [208] |
| *Mouse* | Prp | WT  M337V | Nucleus | NIIs and NCIs TDP-43+, HP, CTF | Reduced  WT = mutant | Gait abnormalities  Tremor  Weight loss | Mutant and WT animals do not differ | [212, 213] |
| *Mouse* | Prp | hTDP-43 WT  A315T  M337V | Homozygous: nucleus and cytoplasm  Heterozygous: mainly nuclear | NCIs pTDP43+, ub+ | Reduced  WT = mutants | Weakness, spasticity , reduced movements  Weight loss  WT = mutants | Degeneration of motor neurons  WT = mutants | [177] |
| *Mouse* | Thy 1.2 (neurons) | hTDP-43 WT | Nucleus | NIIs TDP-43+, FUS +, NCIs TDP-43- | Highest expression: die within 3 weeks, others NR | Growth retardation  Gait abnormalities  Tremor | No apopotosis of motor neurons  Accumulated mitochondria  GEMs abnormalities | [173] |
| *Mouse* | caMKII (hippocampus, cortex and striatum) | mTDP-43 WT | Nucleus | NCIs, ub+, TDP-43+, CTF+ | Reduced | Gait abnormalities  Weakness, spasticity, reduced movements  Impaired learning and memory | Loss of cortical neurons | [187] |
| *Mouse* | Transgene from BAC  endogenous promotor | WT  A315T  G348C | WT: mostly nucleus, mutants: cytoplasmic | NCIs CTF+  Mutant>WT | No premature death | Motor dysfunction (reduced rotarod performance)  Impaired learning and memory | Axonopathy  No motor neuron death | [180] |
| *Mouse* | CaMKII-tTA (Tet-off inducible) | hTDP-43 WT  dNLS | Nucleus | Rare NCIs pTDP-43+, Ub+ | NR | Abnormal limb clasping | Loss of dentate gyrus neurons, axonal loss in corticospinal tract | [89] |
| *Rat* | Transgene from BAC  CAG-tTa | hTDP-43 WT M337V | Diffusely present in nucleus and cytoplasm in WT and mutant | Rare NCIs, TDP-43+ in mutant, ub+, HP, CTF+ | WT: no death  M337V: reduced | WT: normal  Mutant: loss of mobility, limb weakness, paralysis, reduced rotarod performance | Degeneration of motor neurons  in mutant not WT animals | [222] |
| *Mouse* | NA | Knockout | NA | NR | Homozygous: lethal  Heterozygous: normal expression level | Heterozygous: no phenotype | NR | [172] |
| *Mouse* | NA | Knockout | NA | NR | Homozygous: lethal  Heterozygous: normal expression level | Heterozygous: no phenotype | NR | [210] |
| *Mouse* | NA | Knockout | NA | NR | Homozygous: lethal  Heterozygous: normal expression level | Homozygous: not viable  Heterozygous: motor disturbance and muscle weakness | No motor degeneration | [112] |
| *Mouse* | Hb9 (motor neurons) | Knockout  (deletion of exon 2 en 3) | NA | NCIs Ub+ | Reduced | Weight loss  Hind limb clasping  Deficiency in rotarod test | Motor neuron loss |  |
| *Zebrafish* | NA | mRNA  hTDP-43 WT  A315T  G348C  A382T | NR | NR | NR | swimming deficits G348C and A382T > WT and A315T | Shorter axons, excessive branching , no motor neuron death | [99] |
| *Zebrafish* | NA | hTDP-43 WT  A315T | Nucleus | NR | NR | Motor defects | Reduced axon length, aberrant branching | [118] |
| *Zebrafish* | NA | Knockdown  AMO | NR | NR | NR | Swimming deficits | Shorter axons, aberrant branching, no motor neuron death | [99] |
| **FUS** |  |  |  |  |  |  |  |  |
| *C. Elegans* | Prgef-1 (pan-neuronal) | hFUS WT  R521G  R514G  R522G  R525L | WT: nucleus  R521G and R514G: mainly nuclear R522G and R525L: cytoplasmic | Cytoplasmic FUS in insoluable fraction, NCIs, FUS+ | Life span reduced mutants>WT | Motor defects mutants>WT  Cytoplasmic localization correlates woth motor defects | NR | [146] |
| *C. Elegans* | Unc-47 | hFUS WT  FUS S57d | Mutant FUS present in nuclear and cytoplasmic accumulations  WT NR | Mutant FUS in insoluable fraction | Normal | Paralysis  Mutant > WT | Motor neuron degeneration  Mutants > WT | [190] |
| *Drosophila* | Appl (pan-neuronal)  OK371  GMR  Elav-GS (inducible) | hFUS WT | Mutants show increased cytoplasmic localization | Mutants: NCIs ub+ | Appl and OK371: Pupal lethality | Eye: degeneration  Pan-neuronal and motor neurons: locomotor deficits | Mutants more severe phenotype than WT (but WT also affected)  Mutating NES blocks mutant toxic effect | [119] |
| *Drosophila* | GMR  OK 107  OK 371 | hFUS WT  R524S  P525L | WT: predominantly nuclear  Mutants: cytoplasmic inclusions | Mutants NCIs FUS+ | NR | OK 371: locomotion deficits | GMR: retinal degeneration  OK 107: Axonal loss  OK 371: cell swelling and reduced number of boutons  Mutants > WT | [32] |
| *Drosophila* | Elav | hFUS WT  R522G  P525L | WT nucleus, mutants nuclear and cytoplasmic localization | NR | Reduced FUS mutants not WT | Locomotion deficits in mutants not WT | WT: NMJ expansion  Mutants: no NMJ expansion | [203] |
| *Drosophila* | Elav  D42  Act5C | Caz  hFUS WT  R521G  Fus delta 32  Fus delta NES | WT: nucleus  R521G: small increase in cytoplasmic staining  FUS delta 32 en delta NES: largely cytosolic | No NCIs | Reduced  WT = mutants | FUS WT and mutant locomotion deficits not present in delta32 variant | Motor neuron apoptosis  NMJ defects  WT = mutant | [211] |
| *Drosophila* | GMR  Elav | hFUS WT | Nucleus | GMR: no NCIs  Elav: nuclear and diffuse cytoplasmic localization  FUS in insoluble fraction | Elav: reduced | NR | Eye: weak abnormal eye phenotype | [139] |
| *Drosophila* | UAS  GMR  OK371 | hFUS WT  R521C  R518K  and RNA binding mutant (4-FL)  R521C -4FL  R518K- 4FL | WT: nucleus  R521C and R518K: nucleus and cytoplasm  R521C 4-FL and R518K 4-FL: nucleus | Mutants diffusely present in the cytoplasm | UAS mutants: lethal | OK371 mutants: locomotion deficits | GMR: eye degeneration mutants not WT or 4-FL mutant  OK371: decreased brain size mutants not WT and not 4-FL mutants | [42] |
| *Drosophila* | NA | Knockdown Caz (deletion promotor and 58% of caz gene) | NA | NR | Reduced | Reduced eclosion (14%), Locomotion deficits,  Eye phenotype, abnormal genitalia, defects in bristles and wing vein organization | NR | [203] |
| *Drosophila* | Actin5C  Elav | knockdown  using RNAi | Nucleus | No NCIs | Actin: lethal | Neuron-specific: reduced climbing ability in adulthood | MN specific: no motor neuron apoptosis, NMJ: abnormalities: reduced branch length, decreased number of synaptic boutons | [170] |
| *Drosophila* | MS1096 (wings) | knockdown using RNAi | NA | NR | Reduced | NR | Wing growth affected | [211] |
| *Mouse* | Somatic brain transgenic mice, recombinant adeno-associated virus | hFUS WT  FUS R521C  FUS delta14 | WT and R521C: predominantly nucleus  Delta 14: increased cytoplasmic localization | Delta 14: Basophilic and eosinophilic NCIs, FUS+, TDP-43-, PABP+ | Healthy up to 3 months | None up to 3 months | No neuronal loss or degeneration up to 3 months of age | [198] |
| *Mouse* | Prp | hFUS WT | Cytoplasmic mislocalization in homozygous animals | NCIs, FUS+, Ub- | Reduced | Heterozygous: no phenotype  Homozygous: tremor, hind limb paralysis, | Motor neuron degeneration and loss in homozygous animals | [142] |
| *Rat* | Tet-off TRE and tTA system (induced on weaning) | hFUS WT  R521C | Mainly nuclear, diffusely in cytoplasm, mutant affected localization to a minimal extent | NCIs ub+ FUS- | WT: normal  R521C: reduced | WT: no motor phenotype, cognitive decline  R521C: progressive paralysis  Weight loss  Reduced learning and memory | Modest motor neuron degeneration but not loss in motor neurons in mutant not WT  neuronal loss in cortex and hippocampus in mutant and WT | [86] |
| *Mouse* | NA | Knockout | NA | NR | Die shortly after birth | Genomic instability, immunological deficits | NR | [81] |
| *Mouse* | NA | Knockout | NA | NR | Die shortly after birth | Genomic instability, immunological deficits | NR | [115] |
| *Zebrafish* | NA | hFUS WT  H517Q  R521G  R495X  G515X | WT, H517Q, R521G, predominantly nucleus.  R495X, G515X: cytoplasmic | NR | NR | None | None | [25] |
| *Zebrafish* | NA | hFUS WT  R521H  R521C  Sdelta57 | NA | NR | NR | R521C and Sdelta57: no motor phenotype  R521H: reduced touch-evoked escape response | hyperbranched axons | [98] |
| *Zebrafish* | NA | Knockdown AMO | NA | NR | NR | Defect in touch-evoked escape repsonse | Motor neuron degeneration, hyperbranched axons | [98] |
